# Indoor Positioning on Smartphones Using Built-In Sensors and Visual Images

**DOI:** 10.3390/mi14020242

**Published:** 2023-01-18

**Authors:** Jiaqiang Yang, Danyang Qin, Huapeng Tang, Haoze Bie, Gengxin Zhang, Lin Ma

**Affiliations:** 1 Department of Electronic and Communication Engineering, Heilongjiang University, Harbin 150080, China; 2 National Mobile Communications Research Laboratory, Southeast University, Nanjing 210096, China; 3 Department of Electronics and Information Engineering, Harbin Institute of Technology, Harbin 150080, China

**Keywords:** indoor localization, sensors, visual positioning, machine learning

## Abstract

With the rapid development of mobile Internet technology, localization using visual image information has become a hot problem in the field of indoor localization research, which is not affected by signal multipath and fading and can achieve high accuracy localization in indoor areas with complex electromagnetic environments. However, in practical applications, position estimation using visual images is easily influenced by the user’s photo pose. In this paper, we propose a multiple-sensor-assisted visual localization method in which the method constructs a machine learning classifier using multiple smart sensors for pedestrian pose estimation, which improves the retrieval efficiency and localization accuracy. The method mainly combines the advantages of visual image location estimation and pedestrian pose estimation based on multiple smart sensors and considers the effect of pedestrian photographing poses on location estimation. The built-in sensors of smartphones are used as the source of pedestrian pose estimation data, which constitutes a feasible location estimation method based on visual information. Experimental results show that the method proposed in this paper has good localization accuracy and robustness. In addition, the experimental scene in this paper is a common indoor scene and the experimental device is a common smartphone. Therefore, we believe that the proposed method in this paper has the potential to be widely used in future indoor navigation applications in complex scenarios (e.g., mall navigation).

## 1. Introduction

In all aspects of daily life, location service software can be seen everywhere, which brings great help to people’s daily lives, work, and study. However, satellite positioning [1] and base station positioning [2] can only meet the positioning needs of users in the outdoor environment but not indoor positioning needs. In order to meet the urgent need for indoor positioning, many experts and scholars at home and abroad have conducted a lot of research on indoor positioning methods [3] and technologies [4,5,6].

At present, indoor positioning technologies, such as WIFI positioning technology, Bluetooth positioning technology, and RFID (radio frequency identification) positioning technology, have been developed in the field of indoor localization [7]. These technologies are vulnerable to environmental changes, cumbersome data collection, dependence on infrastructure, and other drawbacks, and their applicability is limited.

In recent years, machine vision has developed rapidly, and it brings great convenience to people’s daily lives [8,9]. Unlike other indoor localization methods, vision-based indoor localization usually does not have complex requirements for equipment and the visual variation of buildings is small, so visual indoor localization has more stability. In visual indoor localization, the image retrieval method based on BVW (bag of visual words) has attracted much attention [10,11,12] and clusters the features in images into visual words, making each image representable by visual words. With the development of visual indoor positioning technology, how to meet the practical positioning needs of people’s lives has become the next major research direction in this field. The mainstream solution is to implement visual localization by using smartphone terminals. Currently, most visual indoor positioning techniques do not consider the user’s pose and the user’s photographing direction. In actual localization applications, users can take images in different directions at the same location, while traditional localization methods attribute images in all directions to an image database, which undoubtedly increases the clustering time and localization error for generating visual words.

For this problem, this paper proposes an improved indoor visual localization method based on BVW (IBVW), which is assisted by multiple smartphone built-in sensors for the user’s photographing direction. This method improves the recognition ability of visual words in the process of feature quantification. First, an offline database is constructed based on extracting the SURF (speeded up robust features) feature points of the image. In the online positioning stage, the sensor data of the user’s smartphone is used to identify the user’s photographing direction through the support vector machine, and then the best matching image is obtained through similarity calculations and a re-matching algorithm.

The main contributions of the research are briefly summarized as follows:

(1) A machine learning classifier for mobile phone posture recognition is designed, and a visual localization system based on image retrieval is proposed.

(2) Matching different visual image databases through mobile phone orientation recognition reduces image retrieval time by about 20 percent, improves image matching accuracy, and achieves an average positioning accuracy of 0.4 m.

The rest of this paper is organized as follows: Section 2 introduces related work on visual localization. Section 3 presents the system architecture. Section 4 presents the simulation results and analysis. Section 5 discusses the method proposed in this paper. Finally, Section 6 summarizes the paper.

## 2. Related Work

In the field of indoor positioning technology, past research has focused on related technologies based on received signal strength, such as WIFI positioning technology and Bluetooth positioning technology. WiFi-signal-based positioning technology does not require additional equipment deployment, which has made it very popular in past studies. Wang et al. [13] proposed a WiFi localization method based on fingerprint clustering with Euclidean distance weighting, which solves the problem that the relationship between received signal strength and physical distance is nonlinear. Deng et al. [14] improved the original WiFi localization method by considering the signal attenuation phenomenon caused by the human body. The method considers the influence of the user’s orientation and the smartphone’s carrying position on the signal, which can improve the interference due to the human body. Another typical technology based on the strength of the received signal is Bluetooth location technology; this technology has the advantage of low power consumption and low price. Zheng et al. [15] proposed an efficient beacon location estimation method based on graph optimization, which does not require any dedicated measurement instruments. Pedestrian localization is achieved by collecting inertial readings and readings of received signal strength indications from Bluetooth beacons. However, localization techniques based on received signal strength are very susceptible to a variety of factors, such as multipath fading, building materials, building layout, etc.

Unlike indoor localization techniques based on received signal strength, vision-based indoor localization has no special requirements for the environment. It is not affected by signal fading and multipaths and has higher stability and positioning accuracy in complex indoor environments. At present, the visual indoor positioning technology mainly uses the method of image fingerprint positioning for position estimation; that is, the position information of the camera is stored in the database in advance, and the query image is matched with all the images in the database to obtain the most similar image, thereby obtaining the location estimation of the query image. The most commonly used image fingerprint positioning method is the visual indoor positioning technology based on content-based image retrieval (CBIR) [16], for example, ORB (oriented FAST and rotated BRIEF)-based visual indoor localization systems. The system extracts and stores image features into the database in the offline stage and estimates the target location based on the ORB feature matching results in online positioning. It has been widely used in practice with its fast matching speed and high accuracy.

Although the image-matching-based localization technique can achieve high localization accuracy, the images in the image database increase dramatically as the area to be localized increases, which poses a challenge to real-time localization. To solve this problem, Karim et al. [17] proposed a bag-of-visual-words-based image retrieval method, which uses the SIFT (scale-invariant feature transform) algorithm to feature extraction and description of image features and finally uses the nearest-neighbor classification method to classify new unlabeled images. For the problem that the SIFT feature is a point feature, and the recognition ability of a single local feature is weak, it is affected by the number of visual words in the quantization process. Jia et al. [18] proposed an improved image retrieval method based on bag of visual words.

Xia et al. [19] proposed a simple visual indoor localization method using images, which prepares an image database and its visual vocabulary as well as the corresponding indoor location information in an offline stage by performing feature extraction, description, and clustering on the images. Then, the query image is matched with the image database to obtain the most similar image, and its position is obtained by voting reclassification to obtain the position estimate. Zhu et al. [20] proposed a combinatorial approach to query specific query weights for visual words in images. The emergence of these methods provides technical support for indoor localization technology based on bag of visual words.

However, none of the above methods considers the problem of the user’s photographing direction in practical applications. To solve this problem, this paper proposes an improved image retrieval method based on the bag of visual words. In the database construction stage, different image databases are constructed according to the user’s photographing direction. When positioning, the user’s photographing posture and direction are classified by machine learning technology and the corresponding database is matched according to the photographing direction so as to obtain the position estimation of the query image. In addition, the method only needs a smartphone for indoor positioning.

## 3. System Structure

**Figure 1:**
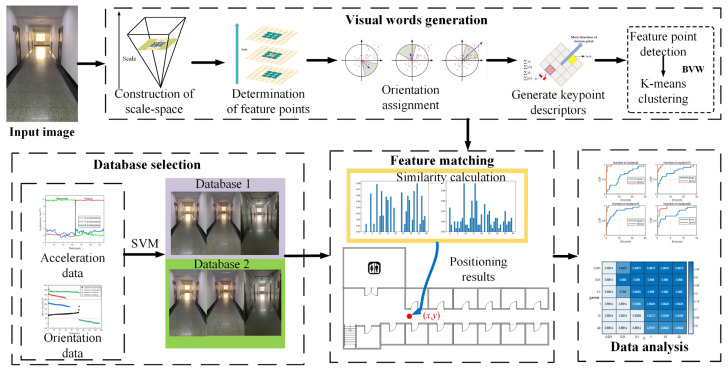
Roadmap of the research.

In this paper, we propose a method for visual indoor localization using multiple sensors. The overall architecture of the method is shown in Figure 1, including the construction of the offline image library and the online positioning stage. In the construction stage of the image database, image acquisition is performed at fixed separation distance in the area of the selected building. In the visual positioning stage, it is necessary to collect the corresponding data from the sensor of the mobile phone, judge the photographing direction of the smartphone according to the data of the mobile phone sensor and match it with the corresponding image database to obtain the final positioning result.

The roadmap of the research is shown in Figure 1.

**Figure 2:**
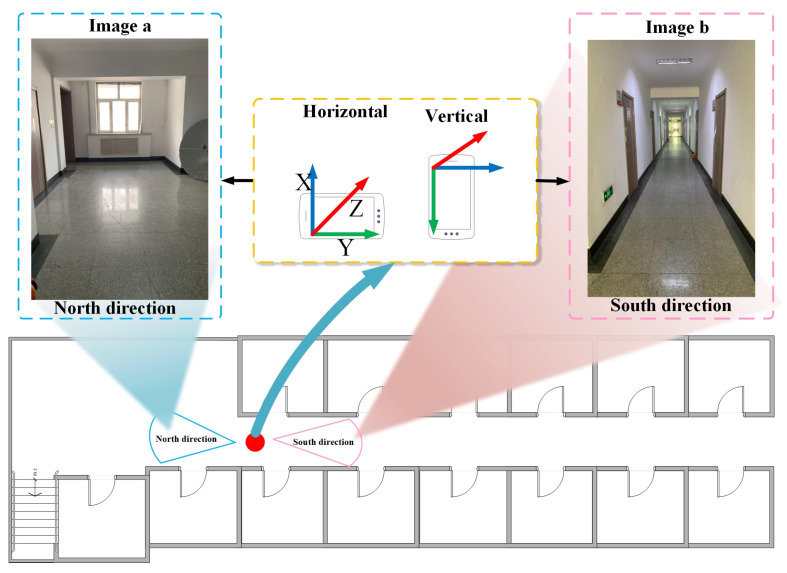
Image acquisition.

The first important task for visual localization is to build an image database. There-fore, an image database needs to be established for the corridors in which the experiments are performed. The image acquisition process in the experimental scene is shown in Figure 2. In selected areas of the experimental scene, image acquisition was performed using a smartphone (iPhone XR, made in Zhengzhou, China)at a distance of 0.6 m with two images per point, where image a and image b are reversed. In this paper, for convenience, we denote the south direction as direction 1 and the north direction as direction 2.

### 3.1. Recognition of User’s Photo Gesture

Most of the current indoor positioning systems based on bag of visual words do not consider the problem of users photographing in different directions. If the images of different orientations are simply classified into the same database, it will not only increase the clustering time but also increase the probability of false matching.

**Figure 3:**
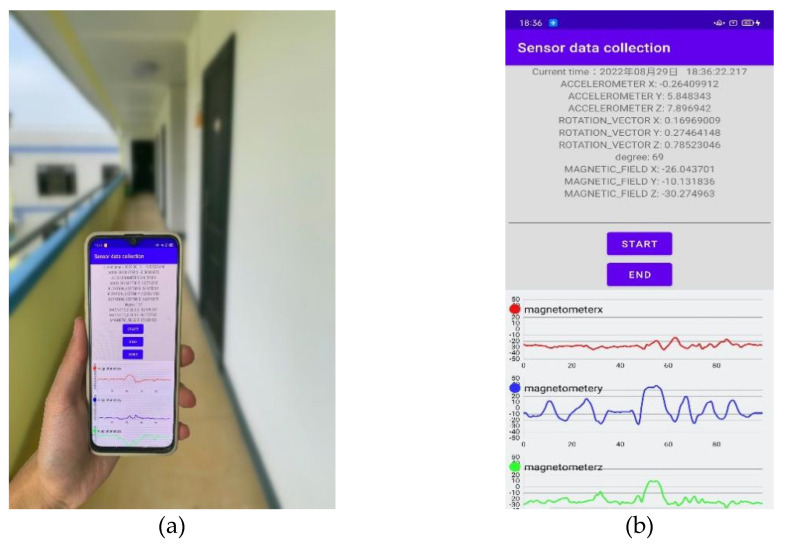
(**a**) Field collection testing; and (**b**) results of data acquisition from smartphone sensors.

Different camera pose recognition will lead to different experimental results. In order to improve the accuracy of indoor visual positioning, this study firstly needs to correctly distinguish the different poses of users using mobile phones to take pictures. To this end, this study divides user modes into two modes, "horizontal photo taking" and " vertical photo taking", and then obtains acceleration data from smartphone sensor data to distinguish these two types of activity modes. The results of the data obtained from the smartphone sensor are shown in Figure 3.

**Figure 4:**
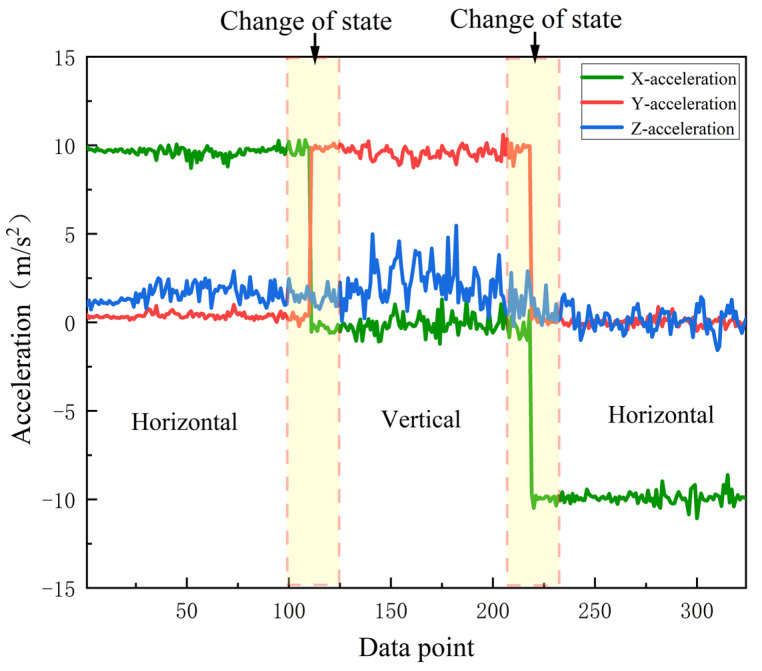
Acceleration of different photo poses in X, Y, Z directions.

Figure 4 shows the changes in the acceleration sensor data along the x, y, z directions under the two user photo-taking attitudes. It can be seen that the acceleration values in the x, y, z directions are very different under different user activity modes. Therefore, this study uses the acceleration feature to distinguish the user’s photo pose.

**Figure 5:**
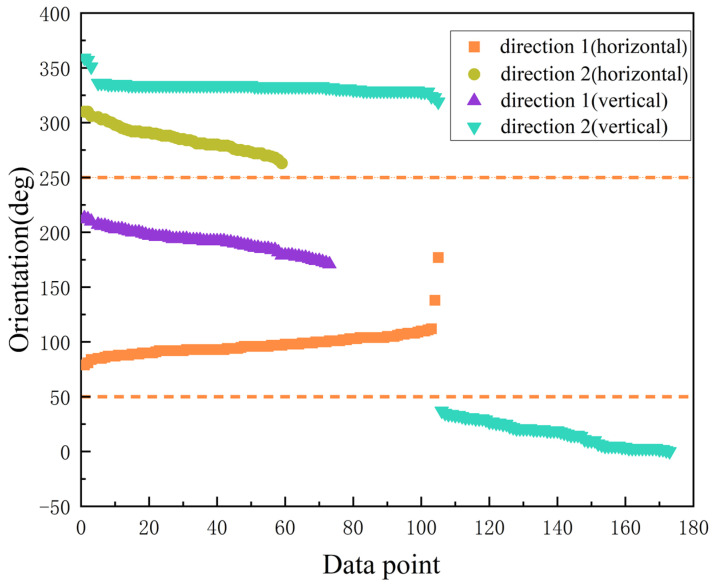
The orientation angle of the phone in different directions.

In our research, we found that the reading of the phone’s orientation meter changes with the direction of our photo when we take a photo for positioning, so the reading of the phone’s orientation meter can be used to distinguish the user’s photo direction. As shown in Figure 5, direction 1 and direction 2 are opposite. The data in different directions are somewhat different. In addition, the function of obtaining the phone orientation meter data directly has been abandoned in Android API 8. According to Google’s official recommendation, we need to calculate the rotation angles ω, φ, γ around the X, Y, Z axes of the device coordinate system based on the accelerometer and magnetometer data and thus calculate the rotation matrix *R*.

(1) $$R=\left[\begin{array}{ccc} \cos\varphi \cos\gamma -\sin\varphi \sin\omega \sin\gamma & -\cos\varphi \sin\gamma -\sin\varphi \sin\omega \cos\gamma & -\sin\varphi \cos\omega \\ \cos\omega \sin\gamma & \cos\omega \cos\gamma & -\sin\omega \\ \sin\varphi \cos\gamma +\cos\varphi \sin\omega \sin\gamma & -\sin\varphi \sin\gamma +\cos\varphi \sin\omega \cos\gamma & \cos\varphi \cos\omega \end{array}\right]$$

After obtaining the rotation matrix R, the orientation meter data are calculated by the SensorManager.getOrientation() function. In further experiments, we found that pedestrians may take pictures in two different postures for orientation (horizontal vs. vertical orientation of the phone), which affects the estimation of the user’s photo direction. This is due to the fact that the various sensor data we get from the phone are based on the device coordinate system, and when we rotate the phone, the sensor data we obtain also change with it. In further research, we found that with the change of pose, the acceleration data changed most significantly when switching between different poses, as shown in Figure 4. Therefore, this paper uses accelerometer data and direction meter data to identify the user’s photo direction.

In order to accurately identify the user’s photo direction, on the basis of collecting a large number of accelerometer data and direction meter data when the user takes a photo, we build a machine learning classifier to recognize user pose patterns [21]. First, *N* samples are collected in the sub-region, and the training sample set is composed of *N* samples.

Each single sample has a one-to-one corresponding label $y_{i}\in \left\{-1,+1\right\}$. The hyperplane can be represented as $w^{T}x+b=0$, where is the normal vector and is the offset. When the equation satisfies Equation (2), the training samples can be correctly classified:

(2) $$\{\begin{array}{cc} w^{T}x+b\geq 1, & y_{i}=+1\\ w^{T}x+b\leq -1, & y_{i}=-1 \end{array}$$

The classification interval is Equation (3):

(3) $$\gamma =\frac{2}{\left\|w\right\|}$$

Finding the optimal hyperplane is actually obtained by maximizing the interval, so the problem of finding the maximal interval to construct the optimal hyperplane can be reconstructed as:

(4) $$\begin{array}{l} \underset{w,b}{\min}\frac{{\left\|w\right\|}^{2}}{2}\\ s.t.y_{i}(w^{T}x+b\geq 1),i=1,2,\ldots ,N \end{array}$$

The minimum problem of such constraints can be solved using the Lagrangian equation by applying the Lagrangian multiplier method to Equation (4):

(5) $$L(w,b,\alpha )=\frac{{\left\|w\right\|}^{2}}{2}-\sum_{i=1}^{N}\alpha_{i}(y_{i}(w^{T}x_{i}+b)-1)$$

After solving, the optimal solution, the optimal offset for dividing the hyperplane, and the optimal normal vector formula for the most dividing hyperplane can be obtained:

(6) $$\alpha^{*}=(\alpha_{1}{}^{*},\alpha_{2}{}^{*},\ldots \alpha_{N}{}^{*})$$

(7) $$b^{*}=y_{i}-\sum_{i=1}^{N}\alpha_{i}{}^{*}y_{i}x_{i}x_{j}$$

(8) $$w^{*}=\sum_{i=1}^{N}y_{i}\alpha_{i}{}^{*}x_{i}$$

According to the optimal solution obtained, the prediction function of Equation (9) can be used to judge the category to which the predicted sample belongs:

(9) $$f(x)=\mathrm{sgn}\left(\sum_{i=1}^{N}\alpha_{i}{}^{*}y_{i}(x_{i}x)+b^{*}\right)$$

When identifying the user’s photographing direction, the "one-to-one" classification method is used repeatedly to identify the user’s photographing direction, and the m(m−1)/2 decisions function can be obtained. When it is necessary to predict the user’s photographing direction, the sensor data are substituted into the decision function, and then the number of winners of all classes is counted. Finally, the class that wins the most is used as the class of the user’s photo direction.

### 3.2. Generation of Bag of Visual Words and Database Establishment

After acquiring the indoor images, we first need to perform feature extraction on the images in the database to ensure the accuracy of matching.

#### 3.2.1. Feature Extraction

In order to speed up the operation, we only perform operations on integral images. We replace the Gaussian filter with a box filter, thus converting the complex image filtering problem into a simple mathematical addition and subtraction problem and speeding up the operation. The integral image is the sum of the grayscale values of all points in the rectangular region formed by the position from the upper left corner $I_{\Sigma }(x,y)$ to any point (x,y) of the image.

(10) $$I_{\Sigma }(x,y)=\sum_{i=0}^{i\leq x}\sum_{j=0}^{j\leq y}I(x,y)$$

Similar to the SIFT algorithm [22], we use the Hessian matrix to detect the feature points in the image. For a point X(x,y), in image *I*, the Hessian matrix with scale σ is:

(11) $$H(X,\sigma )=\left[\begin{array}{ll} L_{xx}(X,\sigma ) & L_{xy}(X,\sigma )\\ L_{xy}(X,\sigma ) & L_{yy}(X,\sigma ) \end{array}\right]$$

where $L_{xx}(X,\sigma )$ is the result of the convolution of image *I* at point X(x,y) with $\frac{\partial^{2}g(\sigma )}{\partial x^{2}}$; the others are similarly calculated.

The box filter and image convolution results are $D_{xx}$,$D_{xy}$,$D_{yy}$; using the simplified resulting images $D_{xx}$,$D_{xy}$,$D_{yy}$, we can perform the following simplification of the Hessian matrix:

(12) $$\begin{array}{c} Det(H)\;\;=L_{xx}L_{yy}-L_{xy}L_{xy}\\ \;\;\;\;=D_{xx}\frac{L_{xx}}{D_{xx}}D_{yy}\frac{L_{yy}}{D_{yy}}-D_{xy}\frac{L_{xy}}{D_{xy}}D_{xy}\frac{L_{xy}}{D_{xy}}\\ \;\;\;\;=D_{xx}D_{yy}\left(\frac{L_{xx}}{D_{xx}}\frac{L_{yy}}{D_{yy}}\right)-D_{xy}D_{xy}\left(\frac{L_{xy}}{D_{xy}}\frac{L_{xy}}{D_{xy}}\right)\\ \;\;\;\;=A\left(\frac{L_{xx}}{D_{xx}}\frac{L_{yy}}{D_{yy}}\right)-B\left(\frac{L_{xy}}{D_{xy}}\frac{L_{xy}}{D_{xy}}\right)\\ \;\;\;\;=\left(A-B\left(\frac{L_{xy}}{D_{xy}}\frac{L_{xy}}{D_{xy}}\right)\left(\frac{D_{xx}}{L_{xx}}\frac{D_{yy}}{L_{yy}}\right)\right)\left(\frac{L_{xx}}{D_{xx}}\frac{L_{yy}}{D_{yy}}\right)\\ \;\;\;\;=\left(A-BY\right)C \end{array}$$

where $Y=\frac{{\left|L_{xy}(1.2)\right|}_{F}{\left|D_{xx}(9)\right|}_{F}}{{\left|L_{xx}(1.2)\right|}_{F}{\left|D_{xy}(9)\right|}_{F}}=0.912≅0.9$. Since the constant C does not affect the comparison of extreme value points, we can proceed further to obtain the approximation:

(13) $$Det\left(H_{\mathrm{approx}\;}\right)=D_{xx}D_{yy}-{\left(0.9D_{xy}\right)}^{2}$$

In order to maintain the scale invariance of the algorithm, we also need to detect feature points in different scale spaces. Therefore, it is also necessary to construct scale pyramids at different scales [23].

**Figure 6:**
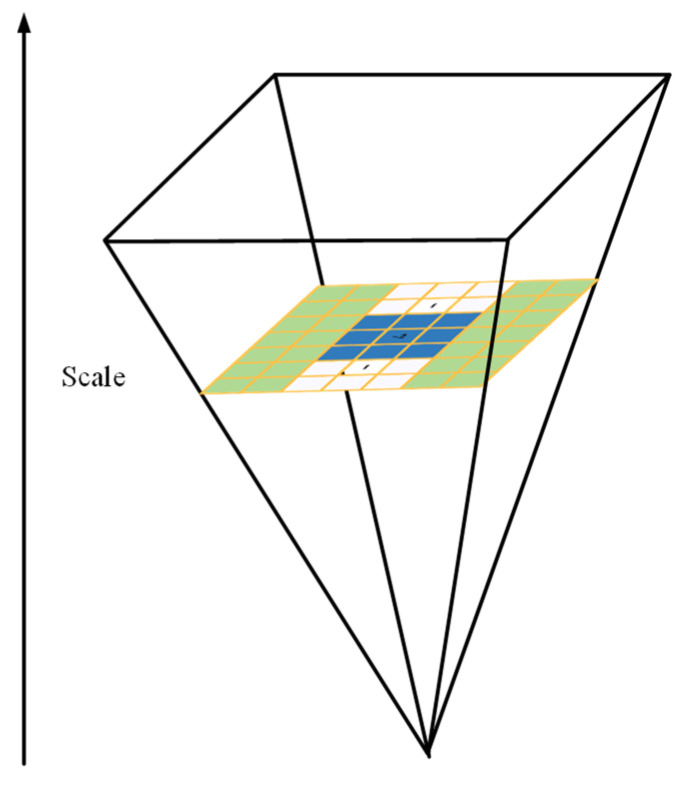
The construction of scale space.

We only change the template size and use gradually increasing size box filters to generate Hessian matrix response images by direct convolution with integral images of different orientations. When constructing the scale space, we keep the original image size constant but change the template size, i.e., the original image is filtered through the template frame as the size changes to construct the scale space. This parallel operation is used to process each layer of the pyramid simultaneously. The downsampling process in the traditional scale pyramid structure is omitted, thus increasing the processing speed, as shown in Figure 6.

**Figure 7:**
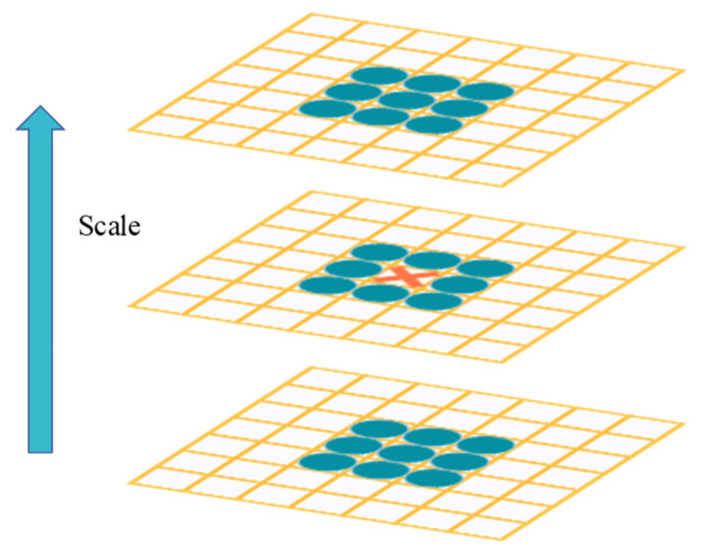
Determination of feature points.

After obtaining the scale pyramid image, the following processing is required in order to select the feature points. If the extreme point is larger than all the extreme points in the comparison range, the point is considered as a feature point, as shown in Figure 7. The location and scale information of the point are recorded.

#### 3.2.2. Feature Point Description

After obtaining the feature points, the principal direction of the feature points needs to be determined in order to ensure the rotation invariance. We use the feature point to be selected as the center of the circle and find the Harr wavelet response values in the x and y directions for all the pixel points within the range in a circular area of radius 6 s, where s is the scale value of the location of the feature point. After calculating the response value of the image, we slide around the feature point with the feature point to be selected as the center of the circle in a sector with an angle of π3 degrees. The sum of the response values in the x and y directions within this range is calculated, and the principal direction of the feature point is obtained.

**Figure 8:**
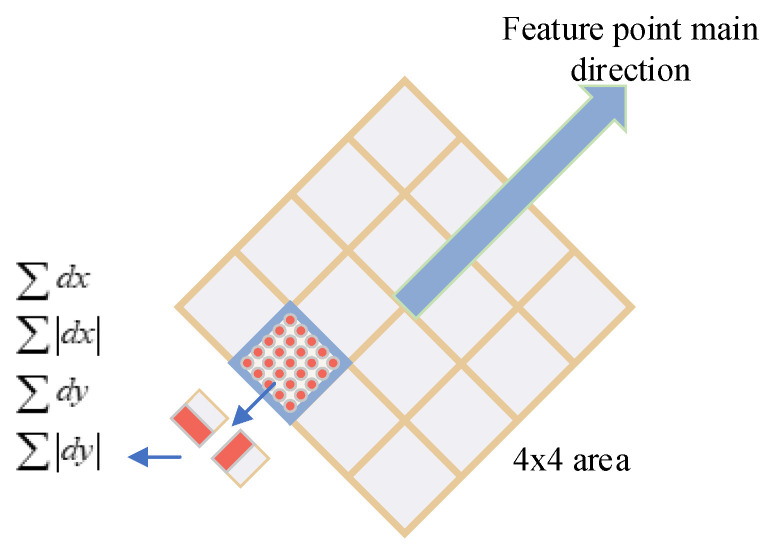
Generating keypoint descriptors.

After determining the main direction of feature points, we can construct feature descriptors. Firstly, we construct the corresponding axes in the main direction of the feature points, and the axes area is a rectangular region of 20 s. We divide this region into sub-regions of 4 s × 4 s. Using a Harr wavelet filter of size 2 s to process the regions, we can obtain the Harr wavelet features for each region. To ensure the robustness of the algorithm, we also need to sum the dx,dy,|dx|,|dy| on each subregion to obtain the feature vector v=(∑dx,∑dy,∑|dx|,∑|dy|). Finally, we concatenate the feature descriptors in all subregions to obtain 64-dimensional feature descriptors, as shown in Figure 8.

**Figure 9:**
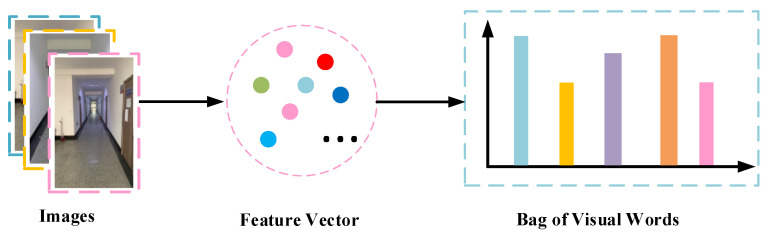
Visual words generation.

Due to the complexity of indoor environments, we will extract a large number of feature points, which poses a great challenge for real-time localization. To solve this problem, we use the K-means clustering algorithm to cluster the extracted feature vectors. Each clustering center is considered as a visual word, thus forming a visual lexicon. After the clustering is completed, each image is stored in the database as a feature histogram. Thus, we convert the complex image matching problem into a simple similarity calculation. For online localization, we only need to calculate the similarity of the histogram between the input image and the database image, which greatly speeds up image matching. The generation process of visual words is shown in Figure 9.

### 3.3. Image Retrieval and Positioning

In the image retrieval stage, the orientation data and acceleration data of the user’s smartphone are first read, and the SVM (support vector machines) algorithm is used to judge the activity pattern of the target user. Then, the corresponding image database is selected for matching after the phone orientation is identified by calculating the similarity between the candidate image and the query image to determine the location of the point to be located.

In this paper, the similarity between the query image and the candidate image is calculated by clustering the feature vectors to form a feature histogram. To improve the accuracy, this paper adopts the normalized term frequency (NTF) weighting algorithm [24] to process the feature histogram. Image *I* consists of a statistical histogram with *k* columns $T_{I}=\left[t_{I1},t_{I2},\ldots ,t_{Ik}\right]$, where $t_{Ii}$ is represented as:

(14) $$t_{Ii}=\frac{n_{Ii}}{n_{I}}$$

where *k* is the number of visual words and $n_{I}$ is the total number of visual words in image *I*. The point of normalization is to remove the difference between image sizes.

In addition to the NTF weighting algorithm, the more classic weighting algorithm is the term frequency/inverse document frequency (TF-IDF) algorithm. In the TF-IDF algorithm, we represent $idf_{Ii}$ as:

(15) $$idf_{Ii}=\log\frac{\left|D\right|}{\left|J\right|}$$

where D is the total number of files, and J is the number of files containing vocabulary $t_{Ii}$.

If a visual word appears very frequently in the feature histogram but very infrequently in the whole database, then the two frequencies are multiplied to obtain a larger weight for filtering the universal visual words and retaining the important ones. Here is the TF-IDF formula:

(16) $$w_{Ii}=idf_{i}\times t_{Ii}$$

The feature histogram of image *I* becomes $T_{I}=\left[w_{I1},w_{I2},\ldots ,w_{Ik}\right]$.

**Figure 10:**
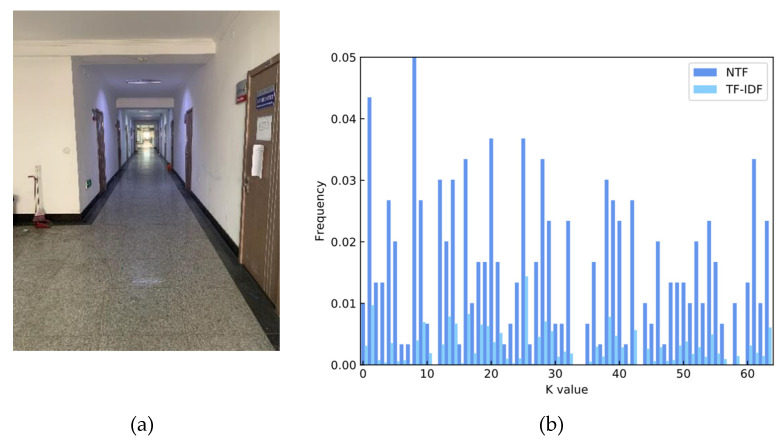
(**a**) Image to be retrieved.; (**b**) image feature histogram using different weighting methods.

The image feature histograms obtained using different weighting methods are shown in Figure 10. From the figure we can see that the TF-IDF method weakens the variability of image features in the indoor environment with sparse feature points.

We use the NTF algorithm to normalize the query image and the image in the database, and then get the histogram. The similarity between the query image and the image in the database is calculated by $\chi^{2}$ [25]:

(17) $$\chi^{2}={\left\|H_{1}-H_{2}\right\|}^{2}=\sum \frac{{\left(H_{1,i}-H_{2,i}\right)}^{2}}{H_{1,i}+H_{2,i}}$$

After calculating the similarity, we sort the images in the database according to the similarity size. The candidate image set is composed by sorting by similarity. The smaller the value of $\chi^{2}$, the higher the similarity between the two images.

The main purpose of the voting scheme is to reduce the amount of computation in the retrieval process and vote on the first three candidate images, as shown in Algorithm 1. If the actual positions of the first three images in the candidate images have a sequential relationship, then the position of the image with the highest similarity is the position of the point to be located. If not, we use the SURF algorithm to detect image feature points and use the FLANN (fast library for approximate nearest neighbors) [26] algorithm for matching, which can calculate the similarity between the candidate image and the query image faster. The image with the most matching points is the best matching image, and the specific process is shown in Algorithm 2.
**Algorithm 1:** Voting Scheme**Input:** all candidate sorted images { $I_{1},I_{2},\ldots ,I_{n}$ }1: Vote for all sorted images { $I_{1},I_{2},\ldots ,I_{n}$ };2: If the top sorted three candidate images { $I_{1},I_{2},I_{3}$ } are associated with the same id;3: Then, the best matching image $I_{best}$
*is*
$I_{1}$;4: Otherwise, the best matching can be acquired using the surf matching Algorithm;**Output:** the best matching image $I_{best}$

In this paper, we use the SURF algorithm for image matching. In order to speed up the matching speed and meet the real-time requirements of user localization, we only use the SURF algorithm to calculate the top 20 images with the highest similarity among the candidate images, as shown in Algorithm 2.
**Algorithm 2:** Rematch Algorithm**Input:** all candidate sorted images { $I_{1},I_{2},\ldots ,I_{20}$ }1: Detect SURF features of candidate image $I_{\theta }$ and query image $I_{query}$;  2: Detect image matching points using FLANN algorithm;3: Calculate the number of matching points, the most is $I_{best}$;**Output:** the best matching image $I_{best}$

## 4. Experimental Results and Analysis

**Figure 11:**
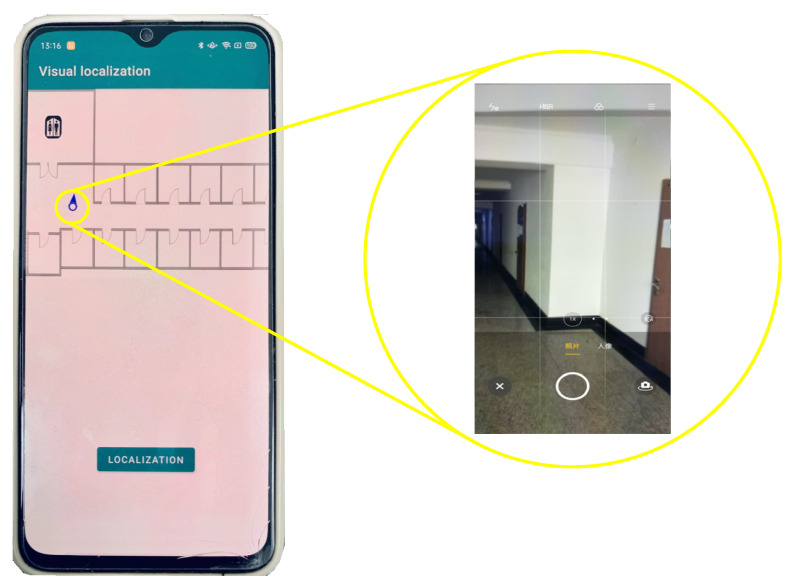
Indoor positioning mobile application.

For the experiments, we used iPhone XR for image acquisition. In the localization test, the test volunteers used OPPO R15X for the localization test. To facilitate the test, we developed mobile application software for photo localization on smartphones and built a localization server using the flask framework, as shown in Figure 11.

We chose the experimental site in the A8 laboratory building: a corridor area of about 33 × 3 m^2^(see Figure 2). The corridor area is open, with no special markers except for some doors and windows. The corridor contains large white walls, and volunteers can perform continuous positioning tests along the corridor. 

### 4.1. Evaluation of User’s Photographing Direction

**Figure 12:**
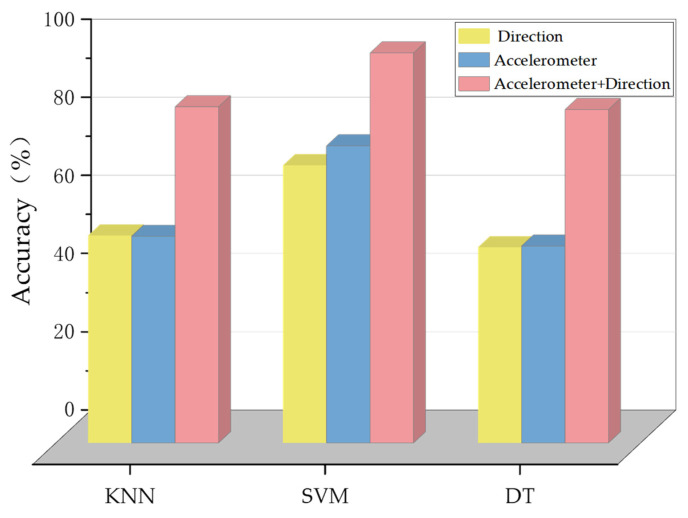
The accuracy of the classification of the user’s photographing direction.

In order to select the optimal classifier parameters for user photo orientation recognition, the performance of SVM with different parameters is analyzed in this paper. The training data include 930 samples of 2 different photo poses, which are sent to the selected classifier for training accordingly. The training vectors include acceleration data, orientation data. Test experiments were performed using 398 samples from the experimental scenes, and the test results are shown in Figure 12, where the SVM kernel function is fixed as a radial basis function (RBF).

The performance of three machine-learning-based classifiers for classifying photo orientation is evaluated. It can be seen from Figure 12 that the accuracy of the SVM classifier is higher than that of the decision tree (DT) and K-nearest neighbor (KNN) classifiers.

**Figure 13:**
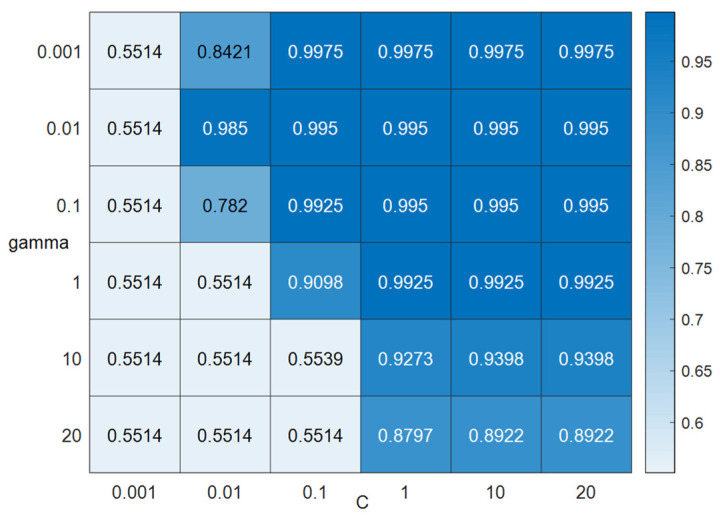
Classification accuracy under different parameters.

As can be seen from Figure 13, when the parameter gamma is 0.01 and the parameter C is 10, the performance of the SVM classifier is the best. Therefore, the parameter gamma used in this paper is set to 0.01, and the parameter C is set to 10 for the machine learning classifier to identify the user’s photographing direction. In addition, it can also be seen from Figure 12 that the classification accuracy using orientation data are slightly lower than that using acceleration data because the orientation data of the mobile phone are largely affected by the attitude of the mobile phone. Additionally, it can be seen that when the orientation data are combined with the acceleration data, higher classification accuracy can be achieved.

### 4.2. Visual Positioning Technology Evaluation

**Figure 14:**
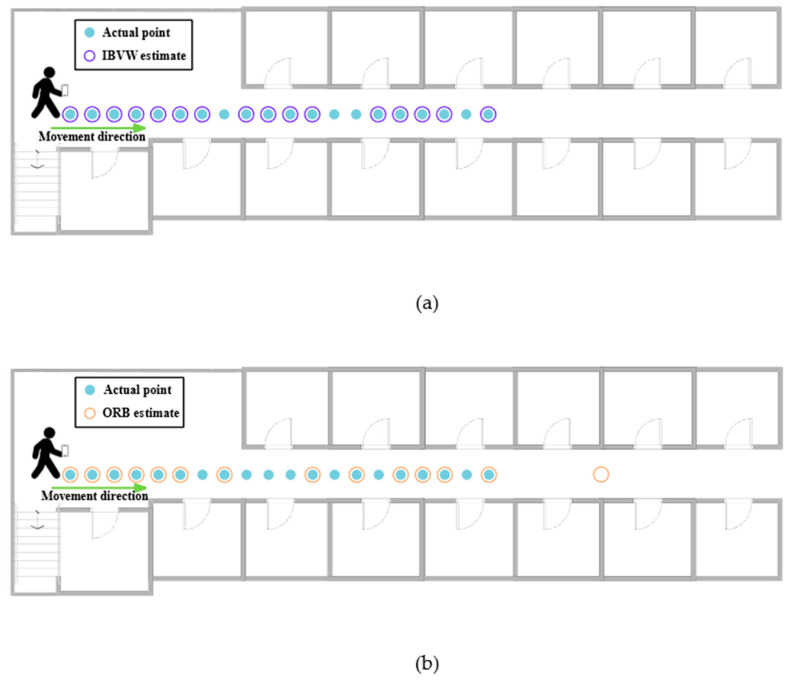
(**a**) Continuous localization map based on IBVW image matching; (**b**) continuous localization map based on ORB image matching.

Localization accuracy is an important index for evaluating localization algorithms. We tested the performance of the proposed algorithm through field experiments. The experimental site was the second floor of the A8 laboratory at Heilongjiang University, and continuous single-point tests were conducted. The localization error of this localization method was analyzed by comparing the actual location with the predicted location and comparing the result with the ORB-based visual indoor localization method. The test results are shown in Figure 14. The blue-green dots represent the preset test points, the purple circles are the predicted locations of the IBVW method (number of clusters = 15), and the orange color circles are the predicted locations of the ORB-based visual indoor localization method. From the figure, we can see that the prediction points of IBVW method are all within the range of continuous test points, which basically match the walking trajectory of pedestrians. The ORB-based visual indoor localization method generally matches the walking trajectory of pedestrians, but there is a problem of the jumping of localization points, and some prediction points are far from the real trajectory of the pedestrians. In the scenes with sparse feature points and a large number of white walls, the proposed method has better performance.

By analyzing the localization errors of the above localization methods, we compared the localization accuracy (the case where the predicted points and the test points completely overlap), the average positioning error (APE), and the standard deviation (SD) of all test points, as shown in Table 1. Through the comparison, we can see that the localization accuracy of the ORB-based method is lower than that of the IBVW method.

**Figure 15:**
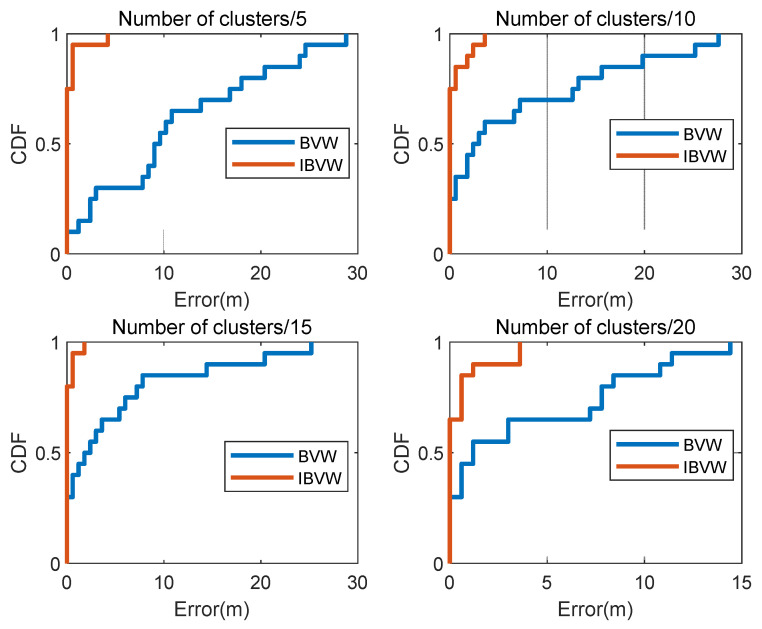
Cumulative distribution function of positioning error.

In addition, the proposed method in this paper is affected by the number of clusters. In order to verify the reliability of the method in this paper, we compare the improved IBVW method with the unimproved BVW method under different numbers of clusters. In the comparison experiments, the BVW retrieval method uses the same database, clusters the extracted feature points using the K-means algorithm, and uses the NTF algorithm to count the feature histograms. For image retrieval, the similarity calculation by Equation (17) is performed to obtain the most similar image, which leads to the position estimation. The CDF (cumulative distribution function) of the two localization algorithms is shown in Figure 15, where the X-axis is the localization error, denoted by “Error”, in meters, and the Y-axis is the cumulative probability of error. When the cumulative probability of error is 1, the value of the corresponding X-axis is the maximum positioning error in this experiment. It can be seen from Figure 15 that the positioning accuracy of IBVW is higher than that of the BVW algorithm, and the algorithm proposed in this paper reduces the average positioning error to less than 1 m. This is because the IBVW method considers the problem of the photographer taking pictures at the same point in different directions and distinguishes the user’s photographing direction through the SVM algorithm, which reduces the number of images to be retrieved, thereby reducing the possibility of false matching. The experimental results show that the proposed IBVW method has higher localization accuracy than the traditional BVW method.

**Figure 16:**
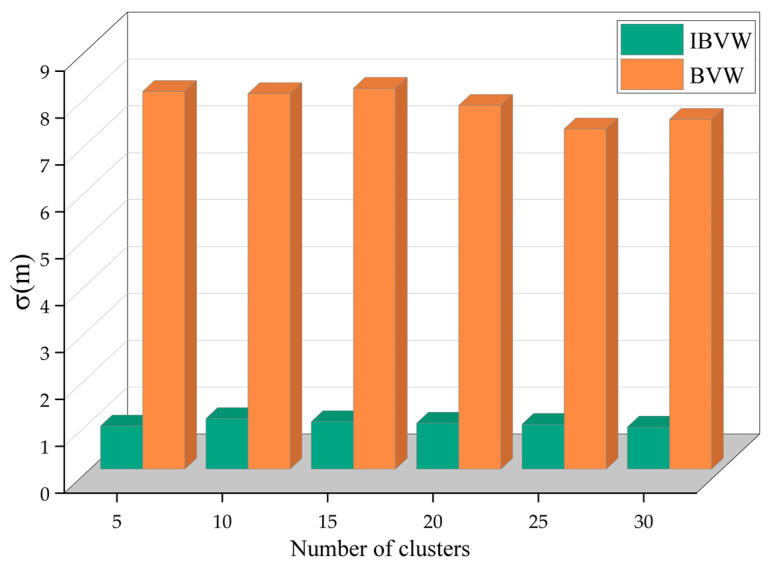
Standard deviation of positioning error.

In the indoor positioning system, in addition to positioning accuracy, we also need to consider the stability of the system. The performance of the two algorithms is compared and analyzed according to the standard deviation, as shown in Figure 16. We use the standard deviation to describe the dispersion of the positioning error. The smaller the standard deviation, the smaller the dispersion of the positioning error. We use Equation (18) to calculate the standard deviation of a set of positioning errors, denoted by σ, in meters. It can be seen from the figure that among the two algorithms, the standard deviation of IBVW is the lowest, and the positioning accuracy is the most stable. The BVW algorithm has the worst stability, with a standard deviation of about 7 m.

(18) $$\sigma =\sqrt{\frac{\sum_{i=1}^{n}{\left(x_{i}-\bar{x}\right)}^{2}}{n}}$$

where σ is the standard deviation, n is the number of test points, $x_{i}$ is the positioning error of the ith test point, and $\bar{x}$ is the average positioning error.

In addition, it is necessary to pay attention to the scale of the visual dictionary. If the *K* value is too small, the visual words will not cover all possible situations, and the word discrimination performance will be poor; if the *K* value is too large, it will be very sensitive to each image feature, require a large amount of calculation, and be prone to overfitting, resulting in low accuracy of the retrieval results.

**Figure 17:**
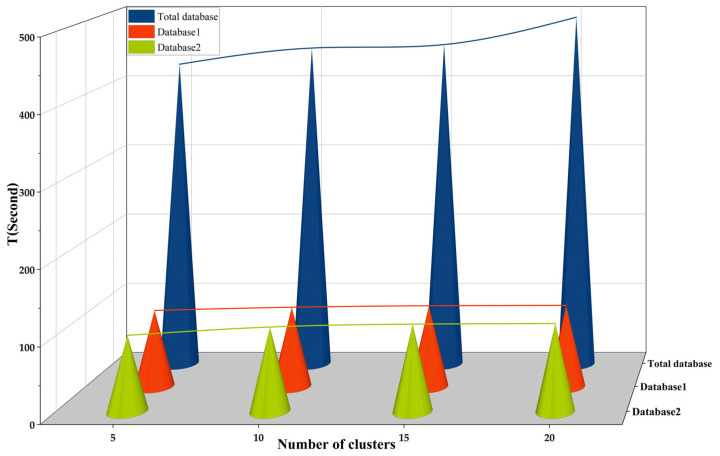
Visual words generation time.

One of the difficulties of visual localization techniques based on BVW is the formation of visual words. As the image database increases, the time to form visual words also increases rapidly, which makes the preliminary work difficult. One of the important metrics for evaluating BVW-based visual localization techniques is visual words generation time. Visual words generation time is the time to transform image features into feature histograms and is expressed in T, in seconds. In this paper, we split the image database into two by taking advantage of the difference in the user’s photo orientation. Under the same experimental conditions, we compared the method used in this paper with the direct clustering method. For the three databases (one original database, two processed databases), the value of *K* was set to 500, 1000, 2000, and 4000 for experiments, and the experimental results are shown in Figure 17.

As can be seen from Figure 17, the total database has more images and extracts more features, so the time consumption for generating visual words is longer. With the method proposed in this paper, the database is divided into database 1 and database 2, so the number of images is reduced and the time consumption is much lower than the time required to generate visual words from the total database. It can be stated that in indoor localization scenarios, larger numbers of images in the image database have a great impact on feature extraction and require longer amounts of time to be consumed. Therefore, for a complex indoor localization scenario, the time efficiency can be effectively improved by the method proposed in this paper with an appropriate reduction of the value of *K*.

## 5. Discussion

The scenes used in this manuscript are corridor areas containing large white walls. The images have sparse feature points and are visually similar in different directions. In my research, I found that rooms in indoor scenes are generally connected by corridors. Users tend to prioritize walking along corridors when looking for the destinations they want to go to (in school buildings, hospitals, etc.). Therefore, this paper uses the SVM classifier to identify the user’s photo direction based on the data from the built-in sensor of the mobile phone during localization, and selects different databases for matching by photo direction, which can reduce the possibility of mismatching. In the open area, we can extract more visual features from the image and the image features with small range of direction changes are not very different. At this time, the effect and advantage of the algorithm proposed in this paper is not obvious. Being able to select a suitable database for matching according to the image orientation will effectively reduce the computational consumption and localization error during image matching. How to distinguish different images more accurately based on the walking direction of pedestrians is one of my follow-up research directions. Here, we hope that the proposed method of visual image database selection based on the built-in sensors of mobile phones in this paper can help experts and scholars in the field of visual indoor localization.

## 6. Conclusions

With the continuous development of smart cities, indoor location services are playing an increasingly important role. The visual-based positioning avoids the use of interference-prone radio signals, so it can achieve accurate positioning in the complex electro-magnetic environment indoors. In the current research, indoor localization technology based on bag of visual words was widely used with high localization accuracy and good anti-interference capability, but it has the problems of low localization accuracy and poor robustness in environments with low background distinction and sparse feature points, such as corridors, libraries, and school buildings. Therefore, this paper proposes a machine-learning-assisted improved BVW method. The method consists of an image database construction phase and an online orientation phase. It mainly carried out the following work:

Firstly, the accelerometer and magnetometer on the smartphone were used to obtain the orientation and acceleration data, and a machine learning classifier was used to identify the user’s photo orientation, reducing the number of images to be retrieved and improving the image retrieval efficiency. In the localization process, the image matching time was reduced from 1 s to 0.8 s, and the localization accuracy achieved 0.4 m, which helped to meet the real-time localization needs of users. In addition, the method proposed in this paper reduced the time of visual word generation by about 52% in the preliminary preparation, which helped to reduce the workload of the preliminary work. Then, the mapped images were matched with the image database, and the similarity calculation, voting scheme, and re-matching algorithm were used to determine the location and improve the overall localization accuracy.

The experimental results show that the proposed method can control the localization error within 1 m and reduce the number of images to be retrieved by 50%, which is a significant improvement to the BVW method and provides a technical guarantee for future indoor localization.

## Figures and Tables

**Table 1:** Comparison of positioning algorithm.

Method	Accuracy	APE	SD
IBVW	75%	0.39 m	0.97 m
ORB-based	55%	1.05 m	3.23 m

## Data Availability

The data presented in this study are available on request from the corresponding author. The data are not publicly available due to privacy.
